# Supplementary material for: Pollution Characteristics and Human Health Risk Assessment of Heavy Metals in Street Dust from a Typical Industrial Zone in Wuhan City, Central China
Source: Int J Environ Res Public Health. 2022 Sep 2;19(17):10970. doi: 10.3390/ijerph191710970 (PMC9518381; doi:10.3390/ijerph191710970)
Supplement: Supplementary file 1 [file ijerph-19-10970-s001.zip › ijerph-1839508-supplementary.pdf]

Supplemental Materials (SM) to

Status, sources, and human health risk assessment of heavy metals in street dust from a typical industrial zone of Wuhan City

Hong Chen[1,2,3], Changlin Zhan[1,2,4*], Shan Liu[1,2], Jiaquan Zhang[1,2], Hongxia Liu[1,2], Ziguo Liu[1,2], Ting Liu[1,2], Xianli Liu[1,2], Wensheng Xiao[1,2]

(1. School of Environmental Science and Engineering, Hubei Polytechnic University, Huangshi, 435003, China;
2. Hubei Key Laboratory of Mine Environmental Pollution Control and Remediation, Hubei Polytechnic University, Huangshi, 435003, China;
3. College of Environment, Zhejiang University of Technology, Hangzhou, 310014
4. State Key Laboratory of Loess and Quaternary Geology, Institute of Earth Environment, Chinese Academy of Sciences, Xi'an 710061, China)

Corresponding author: zhanchanglin@hbpu.edu.cn (C.L. Zhan)

Table S1 Classification criteria of potential ecological risks

Single element potential ecological risk coefficient（E_r^i）		Comprehensive potential ecological risk index（RI）	
≤30	Minor ecological hazard	≤80	Minor ecological hazard
30~60	Medium ecological hazard	80~160	Medium ecological hazard
60~12	Strong ecological hazard	160~320	Strong ecological hazard
120~240	Very strong ecological hazard	≥320	Very strong ecological hazard
≥240	Extremely strong ecological hazard		

Table S2 Parameter values in average daily dose calculation models of heavy metals

Parameter	Physical meaning and unit	Value	
		adult	Child
C	Heavy metal content（mg/kg）	95%UCL	95%UCL
IngR	Frequency of manual-oral intake（mg/d）	100	200
InhR	Respiratory intake frequency（m^3/d）	12.8	7.63
CF	Unit conversion（kg/mg）	1×10^{-6}	1×10^{-6}
EF	Exposure frequency（d/a）	350	350
ED	Exposure duration（a）	24	6
SA	Exposed skin area（cm^2）	1701	899
SL	Adhesion coefficient of skin（$mg/(cm\cdot d)^{-1}$）	0.07	0.2
ABS	Skin absorption factor	0.001	0.001
PEF	Particulate emission factor（m^3/kg）	1.32×10^9	1.32×10^9
BW	middleweight（kg）	56.8	15.9
AT	Mean total exposure time（d）	Non-carcinogenic：ED×365；<br>carcinogenesis：70×365	
